# “Ensure that you are well aware of the risks you are taking…”: actions and activities medical tourists’ informal caregivers can undertake to protect their health and safety

**DOI:** 10.1186/s12889-017-4442-1

**Published:** 2017-05-22

**Authors:** Valorie A. Crooks, Rebecca Whitmore, Jeremy Snyder, Leigh Turner

**Affiliations:** 1 Department of Geography, Simon Fraser University, Burnaby, Canada; 2 Faculty of Health Sciences, Simon Fraser University, Burnaby, Canada; 3 Center for Bioethics and School of Public Health, University of Minnesota, Minneapolis, USA

**Keywords:** Medical tourism, Informal caregiving, Transnational care, Canada, Qualitative, Informational intervention, Caregiver burden

## Abstract

**Background:**

When seeking care at international hospitals and clinics, medical tourists are often accompanied by family members, friends, or other caregivers. Such caregiver-companions assume a variety of roles and responsibilities and typically offer physical assistance, provide emotional support, and aid in decision-making and record keeping as medical tourists navigate unfamiliar environments. While traveling abroad, medical tourists’ caregiver-companions can find themselves confronted with challenging communication barriers, financial pressures, emotional strain, and unsafe environments.

**Methods:**

To better understand what actions and activities medical tourists’ informal caregivers can undertake to protect their health and safety, 20 interviews were conducted with Canadians who had experienced accompanying a medical tourist to an international health care facility for surgery. Interview transcripts were subsequently used to identify inductive and deductive themes central to the advice research participants offered to prospective caregiver-companions.

**Results:**

Advice offered to future caregiver-companions spanned the following actions and activities to protect health and safety: become an informed health care consumer; assess and avoid exposure to identifiable risks; anticipate the care needs of medical tourists and thereby attempt to guard against caregiver burden; become familiar with important logistics related to travel and anticipated recovery timelines; and take practical measures to protect one’s own health.

**Conclusion:**

Given that a key feature of public health is to use research findings to develop interventions and policies intended to promote health and reduce risks to individuals and populations, the paper draws upon major points of advice offered by study participants to take the first steps toward the development of an informational intervention designed specifically for the health and safety needs of medical tourists’ caregiver companions. While additional research is required to finalize the content and form of such an intervention, this study provides insight into what practical advice former caregiver-companions state should be shared with individuals considering assuming these roles and responsibilities in the future. In addition, this research draws attention to the importance of ensuring that such an intervention is web-based and readily accessible by prospective caregiver-companions.

## Background

Informal caregiver burden – or the physical, psychological, emotional, and financial impacts of providing unpaid care labour in a short- or long-term context for an ill, dying, or otherwise in need person on the caregiver – is increasingly recognized as a public health concern [1, 2]. A leading driver of the framing of caregiver burden as a public health issue is the recognition that providing care for others is often a responsibility shared by family members and friends of individuals requiring assistance and is not restricted to the domain of formal health and social care services [3, 4]. For example, recent policy interventions by the Canadian government, such as programs that enable leave from paid work to provide care, have begun to recognize the needs of caregivers as a public health issues, recommending interventions to protect their health [5, 6]. Health research into caregiver burden has typically focused on areas such as the gendered nature of care [7–9], end-of-life care [10, 11], care provided in the home [12, 13], and the dual burden of managing paid work and unpaid caregiving [14–17]. Here we offer a novel perspective on informal caregiving by examining the experiences of the friends and family members who accompany Canadian medical tourists seeking surgery abroad, specifically considering the ways they can protect their health and safety and thus avoid the onset of caregiver burden.

Existing scholarly research on informal caregiving in medical tourism, though minimal, has focused on the emotional experiences of these caregivers [18, 19], their roles in medical tourism from the perspective of destination facility staff [20, 21], the journeys they undertake in traveling for care [22], and the challenges they face while abroad, including language and communication barriers, financial pressures, and emotional strain [23]. In this article we offer new insight into understanding the experiences of this caregiver group by examining the actions and activities that former informal caregivers in medical tourism advise future caregivers to undertake to protect their own health and safety. We refer to these informal caregivers as caregiver-companions in order to acknowledge the full scope of their roles. As we show, Canadians who have served as caregiver-companions recommend health and safety protective actions and activities based on their own lived experiences, and advise that undertaking these steps can reduce caregiver burden and help prospective caregiver-companions limit exposure to specific risks. In examining this issue we apply a public health perspective to the discussion. In particular, we consider the potential of the findings for use in protecting the health and safety of this unique group of short-term informal caregivers via the development of appropriate informational interventions.

Medical tourism is a global health services practice that occurs when individuals travel abroad for the purpose of obtaining privately-purchased medical treatment [24, 25]. Media attention to this health phenomenon is often focused on wealthy patients traveling to the Global South (i.e., countries of lower socio-economic-political development) for medical procedures, although researchers have noted that flows of patients between countries in the Global South are much more common [26–29] than Global North-Global South travel. This paper focuses on the outflows of medical tourists from Canada, and specifically adults who traveled abroad for surgical procedures that they paid for out-of-pocket and who were accompanied abroad by a caregiver-companion. Medical tourists from countries such as Canada, the United States (U.S.) and Western European nations that have good quality health care available at home often share common reasons for travelling abroad for surgery. These rationales include the desire to access procedures not available in their home country, to avoid wait times, to save money on procedures not insured in their home health care systems, and/or to obtain procedures faster than at home [24, 30]. Participation in medical tourism by Canadians is thought to be growing, attracting patients who are increasingly comfortable with international travel, use the internet to identify surgeons and health care facilities, and are inhabiting the role of health care ‘consumer’ despite the presence of a ‘universal’ public health care system at home [31, 32].

Established public health concerns associated with medical tourism include impacts both at the individual and system level across patients’ home countries and destination countries. For example, it is commonly reported that medical tourism can weaken public health care systems by drawing health workers away from under-resourced public sectors in destination countries in order to practice on private-paying foreign patients [24, 33]. Patient and public safety at home and abroad can also be put at risk due to: the potential need to treat complications; possible exposure to antibiotic-resistant infections; patients’ uninformed decision-making; the lack of communication with domestic physicians by clinicians providing care abroad; the lack of regulation in the industry; and the complexity of addressing liability issues [34]. Research on the informal caregiving that takes place in medical tourism has also identified the potential for negative health and safety impacts on caregivers, such as exposure to mental and physical stress [34–36]. The current analysis serves as a novel contribution to the public health-focused medical tourism literature due to our explicit consideration of the potential for the findings to inform the development of an informational intervention aimed at caregiver-companions.

Few public health measures have sought to address the health impacts or risks associated with medical tourism, even through the development of informational tools or surveillance resources [24]. It is for this reason that much medical tourism research has concluded that there is a great need to develop informational resources that can convey important information to potential and intended medical tourists alike, aiding them in decision-making and helping to protect their health and safety [37, 38]. Given the important roles and responsibilities assumed by medical tourists’ informal caregivers as well as their exposure to new (medical) environments abroad [20, 21, 35, 36], we contend that the development of informational tools and other interventions to protect their health and safety is also important. At the same time, little is known about caregiver-companions’ experiences of providing care in this unique transnational context. Thus, a useful starting point for considering how best to protect the health and wellbeing of these caregiver-companions is to consult directly with individuals that have experience in this role. We take this approach here, examining the advice offered by 20 former Canadian caregiver-companions for protecting the health and safety of this group and minimizing caregiver burden. As we document, the advice that they offer highlights specific areas of concern, and identifies potential avenues for public health interventions as well as the content that can populate such interventions.

## Methods

This analysis is part of a larger multi-qualitative method study examining the practice of informal caregiving in medical tourism through consulting with multiple stakeholder groups [20, 21, 36]. The goal of the overall study is to develop an understanding of the roles and responsibilities assumed by caregiver-companions and the risks they face in the practice of transnational informal caregiving. In addition to the interviews reported on here we have also conducted an online survey with Canadian medical tourism facilitators (i.e., agents who privately connect medical tourists with destination facilities and coordinate some travel logistics) and interviews with international patient coordinators (i.e., employees of destination facilities who coordinate care, facilitate information transfer, and ensure medical tourists’ overall comfort).

Between September 2013 and January 2014 we conducted 20 interviews with Canadians who had previously accompanied a medical tourist abroad. Because of the exploratory focus, we targeted speaking with 20 participants in the recruitment period as we believed this would yield sufficient data to identify commonalities and differences across a diversity of care experiences (e.g., in the nature of the relationship between caregiver and care recipient, in destination country visited, in the surgery accessed by the medical tourist, in the extent of care provided). We recruited participants by contacting people who had previously participated in our research on medical tourism, snowball sampling through participants of this study, advertising on Craigslist, and directly contacting people who had been featured in media coverage about medical tourism. Recruitment started after ethics approval was granted by Simon Fraser University’s Office of Research Ethics. After potential participants were identified we provided them with study details, consent information, and also confirmed their eligibility to participate (i.e., they were over the age of 18, had previously accompanied an adult medical tourist abroad for surgery, and were a Canadian resident).

Interviews were conducted by RW by Skype or phone depending on the participant’s preference. Interviews were between 40 and 80 min. At the start of the interview people were informed of their rights as a participant in a research study and were told about how interview transcripts would be stored and how their names and other identifying details would not be disclosed in the study findings. After verbal consent was received, a semi-structured guide was used to prompt discussion. Questions in the guide were exploratory given how little was already known about informal caregiving in medical tourism. Questions probed issues as diverse as: involvement in trip planning; relationship with the medical tourist and the ways in which such relationships changed in the medical tourism context; experiences while abroad, including in the destination facility; and any caregiving challenges that were encountered. The interviews concluded by directly asking participants about advice they have for those occupying the role of caregiver-companion in the future. Interviews were recorded and transcribed verbatim with the exception of one case where the recording device failed and detailed notes were taken instead.

Upon completion of the interviews selected transcripts were independently reviewed in full by RW, VAC, and JS. The purpose of this review was to identify inductive and deductive themes central to the advice participants offered for future caregiver-companions and the experiences that supported or explained why this advice was offered. After we reached agreement regarding the scope of the inductive and deductive themes that had emerged from the data, a coding scheme was developed by RW and VAC in order to organize the data accordingly. RW implemented the scheme using the NVivo qualitative data management program with input from VAC in order to address redundancy. Following this, coding extracts pertinent to the current analysis were reviewed by all authors (RW, VAC, JS, LT) in order to achieve consensus among the team regarding the points of advice offered by participants and the scope of each. Consistent with a thematic approach to analysis [39], these extracts were also considered in relation to the existing medical tourism and informal caregiving literatures to identify confirmation of findings in other studies and the novel aspects of the current analysis. It was through this process that it was agreed that examining the findings through a public health lens, with a focus on the potential to develop an informational intervention based on the advice gathered, would be both novel and beneficial. It is important to note that despite the time that has passed since when the interviews were conducted we have not identified any regulatory or policy changes that would have impacted the trustworthiness of the analysis. In the section that follows we present the analytic findings, offering direct quotes to support the reliability of our interpretation and enhance rigour.

## Results

Of the 20 participants, 13 were men and seven were women. Participants’ ages ranged between 23 and 67, though the majority were over 50 years of age. Half of the caregiver-companions we spoke with traveled abroad with their spouses while the others traveled with a sibling, parent, or adult child. A small minority traveled with a friend. The medical tourists they accompanied obtained a variety of surgical procedures, including: hip or knee replacement, bariatric surgery, cataract surgery, colorectal surgery, and hernia repair surgery. Several of the care recipients went abroad for the experimental chronic cerebrospinal venous insufficiency (CCSVI) procedure for multiple sclerosis (see also [30]).The caregiver-companions and medical tourists travelled to 12 different countries: Aruba, Costa Rica, Egypt, Germany, India, Mexico, the Philippines, Poland, Spain, Turkey, the U.S., and Venezuela. Though the participants had diverse experiences of providing care in medical tourism (see also [35, 36]), their advice to future caregiver-companions that we share in this section reflected the commonalities of their experiences. In the remainder of this section we expand on the five unique yet related themes that examine the points of advice former caregiver-companions have for others planning to assume this role.

### Become an informed health care consumer

Many participants advised prospective caregiver-companions to become informed health care consumers by ensuring that they: (1) understand the procedure the care recipient will be undertaking and its risks, and (2) review information about clinics, doctors, and/or destinations. Participants were almost unanimous in offering this piece of advice, some suggesting that it was useful to start becoming an informed consumer prior to agreeing to take on an informal caregiving role. One participant advised: “*make sure that you gather as much information as you possibly can, research, ask questions before you go.*” To most participants, undertaking such research was actually their first step towards becoming comfortable with the idea of caring for someone who was traveling abroad for medical care. Typically, information was gathered from internet sources. Undertaking online research enabled participants to feel as though they were informed health care consumers. As one participant explained: “*we [participant and the medical tourist] did as much research as we possibly could, you know we were looking up anything online about them [clinics], you know rate your doctor, everything*.” A few participants voiced frustration about the fact that, other than promotional websites, little publicly accessible information was available about medical tourism clinics abroad. Beyond online sources, participants identified previous medical tourists or prior clients of the clinics abroad as another important source of information about procedures, health care facilities, and what generally to expect.

The reason why participants placed great emphasis on becoming informed health care consumers was because it was reported that challenges could arise when caregiver-companions are not well informed about the trip or the medical procedure being accessed abroad. According to participants, being uninformed about key aspects of travel could bring about stress or other negative outcomes. Participants explained that caregiver-companions needed to have a sense of the full scope of the responsibilities they taken on while abroad, from changing wound dressings to locating foods that meet post-operative dietary requirements. They suggested this familiarity came through undertaking research and becoming as informed as possible prior to departure. It was also noted, though, that some caregiver-companions lacked the skills or time to do appropriate research and assess information about medical tourism. One participant commented that:

*…most people don’t have this sort of, well the skills…a lot of people don’t have the determination to do all this research [up front] so maybe a lot of people may end up choosing the wrong type of surgery and the wrong doctor and even though all their intentions are good and they want to be healthy, they have bad outcomes.*

This statement reflected the tension between participants’ emphasis on research and caregiver-companions’ potential inability to access quality information or interpret it effectively. It also drew together a link between medical tourists and caregiver-companions both becoming informed health care consumers, in that a caregiver-companion may be able to augment the information obtained by the care recipient while working together to both become informed health care consumers.

### Assess and avoid exposure to risk

Participants stressed the importance of attentiveness to safety risks, both in the destination at large and specifically in the clinic. They also provided advice concerning how to avoid these threats, which could come from sources as diverse as unscrupulous industry members or unsafe local surroundings. One participant offered the opinion that research was necessary to avoid these potential threats, describing the negative experiences that had been witnessed among or reported by other medical tourists and caregiver-companions while abroad:

*…they had no idea what it was about. They never looked into it. They kind of just paid their money and went and, I mean if they got good results it was good. But some of the doctors that they did go to weren’t very good. And the places they went to weren’t very good [in terms of quality and local safety].*

According to this participant, medical tourists could be at risk of traveling to doctors and facilities that were low-quality, particularly if they failed to do adequate research. It was acknowledged that exposure to such facilities many not only have affected the person undergoing care but also the caregiver-companion, particularly when protocols that ensured safe and clean environments in clinics were lax. However, as noted in the previous section, medical tourists and their caregivers may not have been equipped to conduct research or they could have found it difficult to access reliable information. Thus, as many participants noted, they were vulnerable to potential threats to safety and other risks (e.g., exposure to unclean environments, handling bandages and other medical waste with no protocols for safe disposal, lax patrolling of clinics by security personnel). Awareness of such risks prior to departure was strongly advised: “*ensure that you are well aware of the risks that you are taking going overseas to get a medical procedure done. Yeah, it’s [becoming a caregiver-companion] not a decision that should be taken lightly*.”

Some participants offered warnings for intended caregiver-companions about exposure to unscrupulous and thus risky business practices, including the wide variations in cost between facilities and destinations: “*the end result is these companies [clinics, medical tourism facilitators, etc.] can take advantage of you too, ‘cause the reality is they still have to make money*.” These warnings were framed around concerns for the safety of both caregiver-companions and medical tourists. Participants noted that industry members may not have considered political instability in the destination, local violence, poor clinical outcomes, and/or unsafe clinic environments as they were motivated by profit. The potential for encountering unreasonable prices was also mentioned as a financial risk:

*You have to be aware that you are giving people money and they are going to make money, so there are going to be rogues out there. So you really have to do whatever you can to keep your knowledge of where you’re going and what you’re going to do and don’t go giving money unless you’re absolutely sure of what you’re getting.*

Medically necessary care in Canada was provided with no cost at the point of service (i.e., it is covered through taxation), and so the participants we spoke with were not accustomed to paying directly for surgeries. Engaging in medical tourism therefore added a layer of financial risk to the existing medical risks associated with surgery abroad. In keeping with what was reported in the previous section, participants advised that caregiver-companions should have become aware of the potential for financial burden and have been prepared to make quick, yet financially significant, unexpected decisions while abroad.

### Anticipate the medical Tourist’s care needs

Participants advised that prospective caregiver-companions should have anticipated the care needs of the medical tourist before arriving at the clinic abroad, minimizing the stress involved in providing care and guarding against caregiver burden. Participants who did not adequately do this reported feeling unprepared at times for what was expected of them. It was specifically recommended that intended caregiver-companions should have: (1) been familiar with the medical tourist’s health status, and (2) considered the comfort and emotional support they would need to provide while abroad. Becoming familiar with the medical tourist’s health status before departure and while abroad was important because it was reported that clinic staff often asked caregiver-companions to report on symptom changes, pain levels, and overall improvement. With regard to anticipating the comfort and emotional support needs of medical tourists, participants suggested that intended caregiver-companions may have been able to do things in advance to prepare for this aspect of the caregiving experience. For example, they could have arranged to stay in touch with family at home or have brought a favourite snack or DVD of a beloved television series.

Participants were often quick to mention that from their experiences and observations of others abroad, medical tourists’ care needs were individualized and varied from person to person. Many participants tempered their discussions of this aspect of advice with comments such as: “*It [care needs] would depend on how far developed their condition is*” or “*what level of disability they have*.” Thus, no standard advice was offered with regard to anticipating care needs or determining whether or not they were met. Further, participants noted that caregiver-companions’ roles could be very hands-on and emotionally involved, and that this too should have been anticipated. As one participant explained, caregiver-companions need to be “*comfortable with this person [medical tourist] because it can be very personal of what [care and support] needs to be done”* and that such comfort was important in order to have provided compassionate, intimate, and understanding care.

### Familiarize yourself with important logistics

Drawing on their own experiences of transnational caregiving, several participants advised that caregiver-companions needed to become familiar with travel logistics and anticipated recovery timelines. They advised prospective caregivers to be informed about logistical matters such as arranging flights, ground transportation, and hotel bookings. Doing so enabled smooth transitions between locations and addressing necessary schedule changes should surgical recovery have happened faster or slower than anticipated. One participant explained that caregiver-companions “*have a big role to play in taking care of a lot of the practical details if you do your homework on it, and you probably can’t do too much of that*.” The same participant suggested that talking to prior medical tourists who had undergone the same procedure in the same facility would have been a useful way to get details about anticipated timelines and logistics. This comment was echoed by several other participants who said that caregiver-companions should have gotten *“as much information ahead of time of what’s happening and, you know, the timelines*.” With regard to timelines, participants suggested that caregiver-companions could even have requested information on scheduled appointment times in the clinic in advance of arrival so that they would know what to expect and how appointment schedules may have impacted their own daily routines.

### Protect your health

While most of the advice participants offered was aimed at better supporting medical tourists and taking steps that could indirectly improve caregiver-companions’ health and safety, a few participants noted that it was important for caregiver-companions to take concrete measures that directly protected their own health. However, this was the piece of advice least commonly offered by participants. For example, it was advised that caregiver-companions should have guarded against the physical demands of caregiving by avoiding activities that were overly strenuous while abroad, or planned ahead for such physical strain by becoming familiar with properly performing assistive lifts. Several noted the physical toll that lifting someone with a mobility impairment had in their own experience, particularly in unfamiliar settings such as hotel rooms and/or airplanes. Thus, they drew on their own caregiving experiences in suggesting that prospective caregivers needed to be aware of the physical demands of caregiving. A few others highlighted the need for caregiver-companions to become informed about travel medicine and undertake necessary precautions to protect their health in this regard: “*I think that the most important thing is probably that you have your shots before you go and that the doctors, the GPs [general practitioners] will tell you that, any travel people will tell you that*.” That said, only a few participants discussed actually having undertaken the recommended travel medicine precautions.

## Discussion

In our thematic analysis of 20 interviews with Canadians who had previously accompanied medical tourists abroad for surgery we found that there were several common themes to the advice they had for others planning to assume this role. First, and most commonly, they recommended that caregiver-companions become informed health care consumers by undertaking significant pre-travel research about the destination country and clinic, the medical procedure to be obtained by the medical tourist, and the surgeons. Their recommendation that intended caregiver-companions conduct substantial online research was consistent with the findings of other studies that show that medical tourists heavily rely on web-based information to facilitate informed decision-making [40, 41]. Given the volume of marketing material they encountered, some participants expressed concern about the difficulty they experienced finding reliable information. Second, participants offered advice for assessing and avoiding risks that could have threatened safety or generated stress, whether in the form of interacting with unscrupulous industry members or spending time in unsafe destinations. Not surprisingly, the informal caregiving literature has consistently documented the importance of risk mitigation strategies, such as problem-solving and seeking social support, for avoiding caregiver burden [42, 43]. Third, participants placed a high level of importance on anticipating the medical tourist’s care needs, enabling caregiver-companions to offer both physical assistance and emotional support. Anticipating care needs could assist with identifying unexpected health outcomes or symptom changes, which is something that other studies have shown that health professionals may have looked to informal caregivers for [20, 21]. Fourth, they advised prospective caregiver-companions to have familiarized themselves with important logistical matters, such as travel plans and step-by-step details of medical procedures. Previous research conducted by Casey et al. [20, 21] showed that an important role assumed by caregiver-companions pertains to information gathering and exchange, which would certainly be enhanced by this particular recommendation from our participants. Lastly, a few caregiver-companions advised that others taking on this role protect their own health, such as by visiting a travel medicine clinic prior to departure. However, this was the least commonly mentioned piece of advice, perhaps reflecting the lack of attention participants paid to the impacts of medical tourism on informal caregivers themselves. All too often informal caregivers put the needs of care recipients above their own, which is something that can have a negative impact on their wellbeing and facilitate onset of caregiver burnout [2, 8, 10].

As we noted in the introduction, providing informal care can have negative mental, emotional, financial, and physical health impacts and lead to the onset of caregiver burden [1–3]. The transnational dimension of informal caregiving in medical tourism and the complexities added by providing care in transitional spaces such as hotel rooms and airplanes can amplify some of these potential or realized impacts [36]. Earlier we stated that a goal of this paper is to apply a public health lens to the analytic findings in order to consider the potential for the development of an informational intervention that can be made from the recommendations offered by the participants and their associated activities. A central tenet of public health is the use of scientific findings to develop policies and interventions that promote health and minimize risks to individuals and populations [44, 45]. Given the potential for exposure to health and safety risks in the process of providing informal care to a medical tourist accessing surgery abroad, we contend that it is essential that interventions be developed at the population level (e.g., by health authorities, local/regional/national governments, caregiving advocacy groups, public health officials) that aid in supporting the health and wellbeing of this group. The current analysis provides an important starting point for considering the substantive content of an informational intervention in particular. In the remainder of this section we explore the findings and their implications from this perspective.

Table 1 summarizes the specific actions and activities identified by the participants that can aid in securing the health and safety of future caregiver-companions in medical tourism. Consistent with the findings of Whitmore et al. [35, 36], the participants frequently, but not exclusively, identified these actions and activities based on their own personal experiences of providing transnational informal care as well as others they observed while abroad. Researchers elsewhere have noted that while there is no consensus about the best form of public health interventions to support informal caregivers, it is essential that interventions are tailored to the specific circumstances of particular caregivers [46]. Given the unique features of the combined transnational and short-term contexts of informal caregiving in medical tourism for surgery [36], the actions and activities summarized in Table 1 provide an excellent starting point for considering the content of an informational intervention designed specifically for the needs of this particular caregiver group. Adams et al. [37] provide a precedent for developing an informational intervention for medical tourism stakeholders based on consulting directly with those who had previously engaged in this practice. Specifically, they consulted with former Canadian medical tourists by interview and held focus groups with people in allied sectors (e.g., medical professionals, travel health representatives) to inform the content and format of an information tool intended to aid in the decision-making process. The tool they designed, a single-page pamphlet with a companion website, ultimately incorporated nine specific recommendations for potential medical tourist to consider during this process [37, 47]. In the case of the current analysis, the findings point to 12 specific actions and activities that cross five themes (as summarized in Table 1) that can be included in an informational tool targeting medical tourists’ caregiver companions.

**Table 1:** Summary of Actions and Activities Identified by Participants

Recommendation Theme	Specific Actions & Activities Proposed
Become an informed health care consumer	1. gather information (especially whatever is available online) before and after deciding to become a caregiver 2. ask questions about the procedure and facility (including connecting with former medical tourists) while considering becoming a caregiver-companion 3. understand the types of responsibilities you will have as a caregiver-companion before and after agreeing to take on this role
Assess and avoid exposure to risk	4. research the facility’s quality 5. familiarize yourself with the safety protocols of the facility (e.g., security procedures, hand washing, etc.) 6. arrive prepared to make financially-relevant decisions on behalf of the medical tourist
Anticipate the medical tourist’s care needs	7. familiarize yourself with the medical tourist’s health 8. take steps to anticipate the scope of care activities that will be undertaken (a personalized approach was recommend and therefore no standardized activities were offered)
Familiarize yourself with important logistics	9. become knowledgeable about flight, hotel, and ground transportation bookings 10. talk to former medical tourists who went to the same facility to learn what to expect regarding timelines and other logistics
Protect your health	11. avoid overly physically strenuous activities while abroad (or plan to be prepared for them by practicing patient lifts and transfers before travel) 12. undertake recommended travel medicine precautions (e.g., vaccines)

Following the model of Adams et al. [37], and consistent with the broader literature on knowledge transfer and translation [48], additional research needs to be conducted in order to establish the best messages to be included in an informational intervention designed to protect the health and safety of caregiver-companions, as well as the structure and style of the tool. Further to this process, consideration needs to be given to the most appropriate channels through which intended caregiver-companions can gain access to such an intervention. Becoming an informed patient is challenging enough in a domestic context, but in the context of medical tourism, providing information and facilitating informed decision-making is difficult for many reasons, including the disruption to care continuity [49]. Further, standard pathways for disseminating health-related information, such as between a primary care physician and patient, are often disrupted by medical tourism [50, 51]. The findings of this study provide some assistance in considering how to overcome these challenges and communicate directly to intended caregiver-companions information along the lines of what is summarized in Table 1. Specifically, participants consistently made reference to the importance and value of finding relevant information online. Though they did not point to a specific website as being particularly helpful, their experiences do suggest that a web-based tool is likely to be appropriate. Not only is the internet a valuable tool for gaining access for information about medical tourism [40, 41], but researchers are showing that people are increasingly turning to the web to access all forms of health-related information [48]. Once again, more research is needed so that particular online sites that are commonly visited by intended caregiver-companions can be identified and their appropriateness for hosting an informational intervention assessed. This process should include addressing questions about how to effectively promote such a tool: are medical tourism facilities amenable to hosting an informational tool aimed at supporting the health and safety of caregiver-companions on their websites; which languages should such a tool be made available in and does tool promotion also need to be multilingual; are third party agencies (e.g., public health authorities) willing to serve as hosting agents; and what outreach is available for intended caregiver-companions who have questions about the recommendations? We see the answer to these questions as being particularly important to examine in future research given the prominent way websites, and especially industry websites, were discussed by participants of the current study.

## Conclusion

Informal caregiving can expose people to health risks and lead to the onset of caregiver burden. It is thus not surprising that informal caregiving is increasingly being viewed as a public health issue and that these caregivers are in need of support to protect their health and wellbeing. Here we have examined the experiences of a very unique group of transnational informal caregivers: the friends and family members who accompany medical tourists abroad for surgery to provide care and support. Our analysis of 20 interviews with Canadians who had previously accompanied medical tourists abroad for a variety of surgical procedures revealed five themes around advice for potential caregiver-companions of medical tourists to protect their health and safety. Specifically, they advised becoming an informed heath care consumer, assessing and avoiding exposure to identifiable risks, anticipating the care needs of medical tourists, becoming familiar with important logistics related to travel and recovery, and protecting their own health. This advice forms an important starting point for developing an informational tool to better prepare potential caregiver companions of medical tourists for their role, ideally minimizing exposure to caregiver burden. Future research is needed in order to determine the content of such an intervention and the ideal channels for distribution in order to ensure uptake. We also acknowledge that the perspectives of other stakeholder groups such as medical tourism facilitators, public health experts, and caregiver advocates can augment the advice presented here and thus warrant consideration in the development of such an intervention.
